# Effects of 6-Week Betaine Supplementation on Muscular Performance in Male Collegiate Athletes

**DOI:** 10.3390/biology11081140

**Published:** 2022-07-29

**Authors:** Ming-Ta Yang, Ho-Wei Lin, Chih-Yuan Chuang, Yin-Chun Wang, Bo-Huei Huang, Kuei-Hui Chan

**Affiliations:** 1 Center for General Education, Taipei Medical University, Taipei 110301, Taiwan; yangrugby@tmu.edu.tw; 2 Clinical Research Center, Taipei Medical University Hospital, Taipei 110301, Taiwan; 3 School of Medicine, Taipei Medical University, Taipei 110301, Taiwan; b101107109@tmu.edu.tw; 4 Graduate Institute of Athletics and Coaching Science, National Taiwan Sport University, Taoyuan 333325, Taiwan; giovanie28@hotmail.com (C.-Y.C.); 1060325@ntsu.edu.tw (Y.-C.W.); 5 Charles Perkins Centre, School of Health Sciences, Faculty of Medicine and Health, University of Sydney, Camperdown 2006, Australia; 1040609@ntsu.edu.tw

**Keywords:** bench press, half squat, overhead medicine-ball throw, overhead press, sumo dead lift

## Abstract

**Simple Summary:**

Betaine supplementation has ergogenic potential for exercise performance, but the effect of betaine supplementation in combination with training for 6 weeks is still unclear. The present study aimed to investigate the effects of betaine supplementation on muscular power and maximal strength in collegiate male athletes. The participants in the study received either 5 g/day of betaine or placebo for 6 weeks, during which they maintained their regular exercise training. The overhead medicine-ball throw, countermovement jump, and one repetition-maximum of a bench press, overhead press, half squat, and sumo dead lift by the participants were assessed before and after betaine supplementation. Blood lipids were also analyzed before and after betaine supplementation. We found that receiving 5 g of betaine supplementation daily during the 6-week preparatory period had extra benefits on the power of the upper body and maximal strength on the half squat and overhead press. Betaine seems to be a useful nutritional strategy to improve and maintain performance during 6-week preparatory periods in collegiate athletes.

**Abstract:**

The purpose of this study was to investigate the effects of 6-week betaine supplementation during a preparatory period of collegiate athletes on muscular power and strength. Sixteen male collegiate athletes received 5 g/day of betaine (betaine group, n = 9) or carboxymethyl cellulose (placebo group, n = 7) for 6 weeks. All participants engaged in their regular training during the experimental period. The overhead medicine-ball throw (OMBT), countermovement jump, and maximal strength (one repetition maximum, 1-RM) on the bench press, overhead press, half squat, and sumo dead lift by the participants were assessed before and after betaine supplementation. Blood lipids were also analyzed before and after betaine supplementation. After supplementation, there were no significant differences between betaine and placebo groups on any variables. Compared to presupplementation, the performance of OMBT and 1-RM of overhead press and half squat in the betaine group had significantly improved (*p* < 0.05). By contrast, no significant differences were observed in the placebo group before and after supplementation. Blood analysis revealed no negative effect on blood lipid profiles. Betaine seems to be a useful nutritional strategy to improve and maintain performance during 6-week preparatory periods in collegiate athletes.

## 1. Introduction

Betaine is a natural compound extracted commercially from *Beta vulgaris* [1,2]. It is present in animals, plants, and microorganisms [3]. Betaine levels are high in shellfish, beetroot, spinach, and wheat [4,5]. Ross et al. [6] stated that cereal foods account for approximately 60% to 67% of betaine in Western diets and 20% to 40% of betaine in Southeast Asian diets. The betaine content of food is affected by various cooking methods, with significant losses (60% to 80%) occurring during boiling [7]. Previous research has revealed that consuming 9–15 g of betaine daily is safe for human adults [8]. As early as the 1990s, betaine supplementation was utilized in animals to investigate the effect of betaine on growth [9,10,11] and disease prevention [12]. Betaine can be converted to N,N-dimethylglycine, altering substrate utilization and reducing lactate accumulation during exercise [13]. Warren et al. [13] further investigated this in mature horses based on betaine being converted to N,N-dimethylglycine, which may alter substrate utilization during exercise. The study revealed that betaine supplementation (80 mg/kg BW/day) for 14 days significantly lowered plasma lactate and free fatty acid concentrations elevated by exhaustive exercise [13]. Therefore, betaine supplementation may have potential ergogenic effects on exercise performance. Among many physical fitness components, muscular strength and power are key factors to overall athletic performance [14,15]. Several studies have used muscular strength and power as variables of exercise performance.

The mechanisms by which betaine may be ergogenic on exercise are not fully understood [16]. Betaine has the properties of regulating organic osmolytes and protecting the functioning of cells and mitochondria [2]. Moreover, betaine can donate a methyl group for converting homocysteine to methionine and then synthesize to creatine in skeletal muscle [17,18]. During high-intensity exercise, creatine can rapidly replenish phosphocreatine (PC) and adenosine triphosphate (ATP), consequently serving as a rapid source of energy to enhance exercise power [19,20]. Studies have indicated that betaine supplementation may enhance recovery between training sessions by protecting against protein denaturation, promoting the secretion of insulin-like growth factor 1 [21,22] and growth hormone [22], as well as decreasing cortisol concentration after an acute exercise session [22]. Furthermore, betaine supplementation increased fasting testosterone levels and the testosterone/cortisol ratio and prevents an increase in proinflammatory cytokines during a 14-week competition period [16,23]. Therefore, betaine supplementation may have ergogenic effects on muscular performance of strength and power via maintaining cell completeness, increasing creatine level, and positive responses of the metabolic hormones. Previous human studies provided 1.25 g of betaine mixed in 240–300 mL of sports drink twice per day for 7 consecutive days [24], 10 days [25], or 14–15 days [17,18,26,27] to investigate the effects of betaine supplementation on exercise performance. Some studies reported that betaine supplementation would not enhance exercise performance [25,26,27]. Moreover, the participants in those studies are untrained or recreationally trained individuals [17,18,24,25,26,27,28,29]. Previous studies reported that 2 g/day for 10 days and 2.5 g/day for 14 days of betaine supplementation does not affect strength or power performance in untrained subjects [25,27]. Therefore, supplementation for longer or another betaine supplementation strategy should be discussed. 

In recent years, numerous studies have investigated combined nutritional supplementation with exercise or training programs to reinforce the benefits of training. A study indicated 14 days of betaine supplementation significantly increased the total repetitions and volume load in a 10-set bench press protocol but not improved the performance of vertical jump and leg press [27]. Therefore, supplementing betaine with training may enhance the quality of training and consequently enhance performance. Studies indicated that betaine supplementation (2.5 g/day) for 6 weeks associated with a regular training program or CrossFit training would not improve muscular power and strength in recreationally strength-trained men or regular exercisers [28,29]. Similar results were presented in the studies of 2.5 g/day betaine supplementation combination with resistance training for 9 weeks in collegiate women [30] and 2 g/day betaine during a 14-week competition period in young professional soccer players [31]. However, in the study of 6-week CrossFit training, maximal strength (one repetition maximum, 1-RM) on back squats significantly increased compared to presupplementation, but the strength in the placebo group did not change [29]. The inconsistent results may be due to the training load/volume: betaine supplementation may enhance the stimulation of training and consequently enhance muscular performance. Previous research indicated that consuming 6 g per day of betaine for at least 4 weeks elevated plasma betaine concentration [32]. Therefore, higher dosage of betaine supplementation for a shorter term of 6 weeks may be a practical strategy for athletes, though the benefits on muscular performance in well-trained collegiate athletes remain unclear.

Betaine supplementation increases homocysteine metabolism through betaine donating a methyl group to transmethylate homocysteine, forming methionine [32]. A systematic review reported that increased plasma levels of total homocysteine were a risk factor for cardiovascular disease [33], whereas an average daily intake of 131 mg of betaine may decrease blood homocysteine levels [6]. Long-term betaine supplementation (6 g/day for 12 weeks) decreased plasma homocysteine concentrations, but increased low-density lipoprotein cholesterol (LDL-C) concentrations [34]. Summarizing the results of Schwab et al. [34], consuming less than 6 g of betaine per day for less than 12 weeks may not exert a negative effect on blood lipid levels. Moreover, a previous study showed a single dosage of 5 g betaine supplementation could not improve acute sprint performance in male runners [35]. More studies to discuss effects of 5 g betaine supplementation for more than 4 weeks are needed.

In our study, we recruited well-trained collegiate athletes, because few studies on betaine supplementation on power and strength performance in this population have been conducted. Therefore, we hypothesized that supplementing 5 g/day of betaine for 6 weeks in male collegiate athletes during their preparatory period would effectively enhance their muscular power and 1-RM.

## 2. Materials and Methods

### 2.1. Participants

An a priori power analysis was performed using G*Power software (v.3.1.9.2). For a 2 × 2 two-way mixed-design analysis of variance (ANOVA), with an effect size of 0.4, an alpha error of 0.05, and a power of 0.80, the total number of subjects was calculated to be 16. Twenty male collegiate taekwondo and wushu athletes were recruited to participate. The exclusion criteria were individuals with diabetes and cardiovascular, renal, liver, or autoimmune diseases; with acute or chronic muscular-skeletal injuries; or who had consumed long-term or daily doses of anti-inflammatory medications or nutritional supplements within the past month. Participants were assigned to either the betaine or placebo group according to their 1-RM half squat. Four participants dropped out of the study due to acute sports injuries during regular training and two subjects refused the blood sampling. Therefore, 16 subjects (n = 9 in the betaine group and n = 7 in the placebo group) completed the intervention of supplementation and performance tests, and the blood lipid profile was analyzed with 7 subjects in each group. 

A height scale was used to measure the body height of the participants who were barefoot to the nearest 0.1 cm. The participant’s body weight and body fat percentage were measured using a bioelectrical impedance instrument (InBody 3.0, Biospace, Seoul, Korea) with standard body composition measurement methods. Table 1 presents characteristics of participants in the two groups. There were no significant differences between the groups in age, body height, body weight, or body fat percentage. The study was approved by the Institutional Review Board of Fu Jen Catholic University, New Taipei City, Taiwan (IRB number C106067).

Before participation, the participants provided written informed consent and completed health-related questionnaires. A week before the trial, they were required to refrain from taking any nutritional supplements or medicines and were required to avoid alcohol or caffeine and maintain a regular diet and training regimen. Furthermore, they were instructed to remain their daily dietary pattern and to avoid foods rich in betaine (including, beetroot, spinach, wheat bran, amaranth grain, wheat grain, sweat potato, and shellfish). According to our previous study [36], this dietary strategy can effectively prevent changes in blood betaine concentration caused by food consumption.

### 2.2. Experimental Approach

Two weeks before the experimental session, the participants attended a familiarization session designed to prevent negative effects or sports injuries caused by unfamiliarity with the exercise test. On the first day, before the experiment, overhead medicine-ball throw (OMBT) and CMJ tests were conducted. Subsequently, on the second day, 1-RM of the bench press, overhead press, half squat, and sumo dead lift was evaluated, and propulsive velocity was assessed during each exercise. Three days later, the participants started consuming 2.5 g of betaine or carboxymethyl cellulose twice daily for 6 weeks in a double-blind manner.

This study was conducted during the preparatory period for athletes, and the participants maintained their regular training program, which included sport-specific skills training (2.5 h/day, 5 days/week) and strength and condition training (twice per week). The program of strength and condition training included squat jumps, hang clean pulls, reverse push-ups, pull-ups, and velocity-based resistance training. Each velocity-based resistance training session comprised three sets of half squats, sumo dead lifts, bench presses, and overhead presses with loads ranging from 70% to 95% 1-RM. Repetitions were stopped when a 20% velocity loss had been reached in each exercise set [37]. The participants were also asked to refrain from excessive training outside their sports training.

Approximately 200 μL of blood was collected from participants’ fingertips to analyze the levels of triglyceride (TGs), total cholesterol (TC), high-density lipoprotein cholesterol (HDL-C), and LDL-C on the day before the first supplementation and the morning after the last training. Three days after the last supplementation, we conducted the same test procedures that were conducted before supplementation. 

### 2.3. Overhead Medicine-Ball Throw Test

The OMBT test was conducted to evaluate the muscular power of the upper body, as described in our previous study [38]. After low-intensity aerobic exercise followed by light stretching exercise for warm-up, the participants executed three OMBTs with a 3–5-min rest interval, and the longest distance was used for analysis. During the OMBT test, the participants brought a 3 kg medicine ball back behind the head and were instructed to throw the medicine ball as far forward as possible while standing on a line with their feet slightly apart. The subjects were not allowed to move their feet during the test. 

### 2.4. CMJ Test

After performing low-intensity aerobic exercise at a comfortable pace followed by lower-limb light stretching exercise for warm-up, the participants underwent two CMJs with a 3–5-min rest interval, and the highest height was selected for analysis. During the CMJ test, the participants jumped on a force plate (Kistler type 9284, Kistler AG, Winterthur, Switzerland) with their hands always on their hips, and downward countermovement was performed until the knee angle was approximately 90°.

### 2.5. Prediction of the Load–Velocity Relationship and 1-RM

The protocol described by Dorrell et al. [39] and Morán-Navarro et al. [40] was utilized to predict the 1-RM of bench press, overhead press, half squat, and sumo dead lift. In brief, the participants jogged for 5 min on a treadmill before performing upper/lower-limb light stretching exercises and two light-resistance warm-up sets. After 1 min rest, the participants executed a full range of motion with a 40–95% load of the predicted 1-RM. During the prediction process, three attempts were executed for light (40% 1-RM), two for medium (70% and 80% 1-RM), and one for heavy (85%, 90%, and 95% 1-RM) loads. The interset rest was 3–5 min. Thereafter, the load was individually adjusted in smaller increments to precisely determine the 1-RM strength. Furthermore, during each incremental load, a linear positional transducer (GymAware Power Tool, Kinetic Performance Technologies, Canberra, Australia) was attached to the barbell for calculating and recording the mean propulsive velocity, and the load–velocity relationship was established for each participant. During the experiment, the load (percentage of 1-RM) was converted to propulsive velocity, which was then applied to strength and condition training.

### 2.6. Blood Sampling and Analysis

On the day before the first supplementation and the morning after the last supplementation, participants rested for 10 min in a seated position, and approximately 200 μL of blood from the fingertips was collected into a heparinized capillary tube using an automatic lancet device. Blood samples were centrifuged for 3 min at 3000 rpm. Subsequently, 10 μL of plasma was pipetted to Fuji DRI-CHEM slides of TGs, TC, and HDL-C (Fujifilm, Tokyo, Japan) and measured using the dry multiplayer analytic slide method in a biochemistry auto analyzer (FUJI DRI-CHEM 4000i; Fujifilm, Tokyo, Japan). The concentration of LDL-C was calculated using the Friedewald formula: LDL-C = TC − HDL-C − (TGs/5) [41].

### 2.7. Supplementation

Participants in the betaine group received 99% pure betaine powder (Twinlab Corp., Hauppauge, NY, USA), whereas the participants in the placebo group received carboxymethyl cellulose (food-grade powder, GreenYoung Co., Taichung, Taiwan). The participants consumed 2.5 g of their respective supplements twice daily (30 min postbreakfast and 30 min postdinner). The supplement powder was encapsulated in 500 mg capsules, and the participants ingested five capsules at once with 200 mL of water. The supplements consumed by both groups were identical in color and taste.

### 2.8. Statistical Analysis

Statistical analyses were performed using SPSS version 20.0. The data are expressed as means ± standard deviation. An independent-sample *t*-test was used to compare the subjects’ characteristics between the groups. A 2 (groups: betaine and placebo) × 2 (time points: pre- and postsupplementation) two-way mixed-design ANOVA was used to compare the variables of muscular performance. When a main effect for time was observed, post hoc tests were analyzed by the paired *t*-test. To evaluate the effect size, we calculated the Hedge’s g effect sizes for continuous data, which included a correction for small-sample bias. The Hedge’s g values were interpreted as small (0.20–0.49), moderate (0.50–0.79), and large (≥0.80) [42]. The significance level was set at *p* < 0.05.

## 3. Results

### 3.1. Effects of Betaine Supplementation on the Muscle Power of the Upper and Lower Body

**Figure 1:**
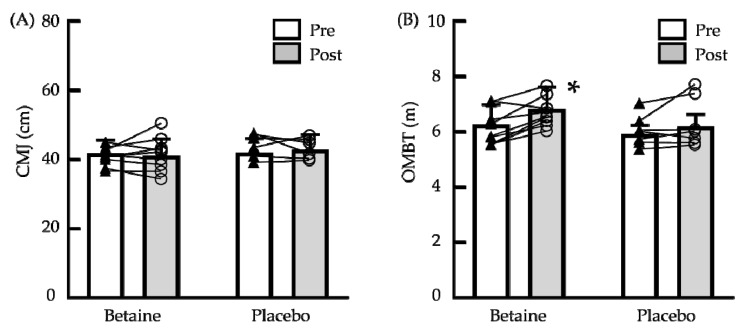
Changes in the muscle power of the upper and lower body before and after 6 weeks of betaine supplementation. (**A**) Overhead medicine ball throw (OMBT); (**B**) countermovement jump (CMJ). Pre: before supplementation; Post: after supplementation. The bars indicate means ± standard deviation. ▲: Individual presupplementation values; ○: Individual postsupplementation values; *: Significant difference from Pre (*p* < 0.05).

Figure 1 illustrates the outcomes of the muscle power of the upper and lower body, as determined by the OMBT and CMJ tests. After supplementation, no significant differences (as indicated by the OMBT and CMJ tests) were observed between the betaine and placebo groups. However, there was a significant main effect for time on the OMBT (*p* = 0.003). Compared to presupplementation, the performance on the OMBT in the betaine group significantly improved (*p* < 0.05; Hedge’s g = 0.75), indicating a moderate effect size.

### 3.2. Effects of Betaine Supplementation on Maximal Muscular Strength

**Figure 2:**
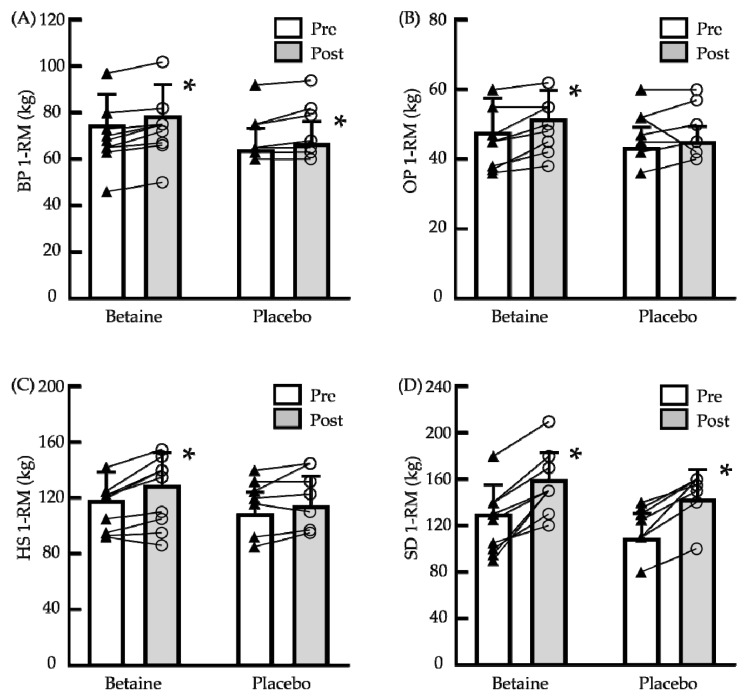
Changes in the one repetition maximum (1-RM) before and after 6 weeks of betaine supplementation. (**A**) Bench press (BP); (**B**) overhead press (OP); (**C**) half squat (HS); and (**D**) sumo dead lift (SD). Pre: before supplementation; Post: after supplementation. The bars indicate means ± standard deviation. ▲: Individual presupplementation values; ○: individual postsupplementation values; *: significant difference from Pre (*p* < 0.05).

Figure 2 illustrates the outcomes of the 1-RM of bench press, half squat, overhead press, and sumo dead lift. After supplementation, there were no significant differences between betaine and placebo groups on the 1-RM strength. There were significant main effects for time on the 1-RM of bench press, half squat, overhead press, and sumo dead lift (*p* = 0.001, 0.003, 0.028, and 0.001, respectively). Compared to presupplementation, the 1-RM of bench press, half squat, overhead press, and sumo dead lift in the betaine group significantly increased (*p* < 0.05; Hedge’s g = 0.30, 0.43, 0.50, and 1.23, respectively). The 1-RM of bench press and sumo dead lift in the placebo group were also significantly increased (*p* < 0.05; Hedge’s g = 0.28 and 1.45, respectively). The results showed that betaine supplementation had small and moderate effect sizes on 1-RM of half squat and overhead press, respectively. Moreover, the regular training of participants had small and large effect sizes on 1-RM of bench press and sumo dead lift, respectively.

### 3.3. Effects of Betaine Supplementation on Blood Lipid Profile

Table 2 illustrates the outcomes of the concentrations of TGs, TC, HDL-C, and LDL-C after 6 weeks of betaine or placebo supplementation. 

After supplementation, there were no significant differences between betaine and placebo groups on the blood profile. There were significant main effects for time on the concentrations of TC and LDL-C (*p* = 0.001 and 0.004, respectively). Compared to presupplementation, the TC concentration in the betaine group significantly decreased (*p* < 0.05; Hedge’s g = 0.91). Moreover, the concentrations of TC and LDL-C in the placebo group significantly decreased (*p* = 0.003 and 0.024; Hedge’s g = 1.17 and 0.87, respectively). The results showed that 5 g/day of betaine supplementation in collegiate athletes of Division I during the preparatory period had no negative effect on blood lipid profiles.

## 4. Discussion

Based on the properties of betaine to maintain cell completeness [2], positive responses of the metabolic hormones [21,22,23], and its ability to donate methylene groups for creatine synthesis [18], we hypothesized that 5 g betaine supplementation during the 6-week preparatory period would effectively enhance muscular power (OMBT and CMJ) and strength (bench press, overhead press, half squat, and sumo dead lift) in male collegiate athletes. To the best of our knowledge, this study is the first to examine the effects of high-dose betaine supplementation on muscular performance in collegiate athletes. Although there were no significant differences between betaine and placebo groups in any variables, compared to presupplementation, the performance of OMBT, 1-RM of overhead press and half squat in the betaine group significantly improved (*p* < 0.05). Our findings indicate that 5 g of betaine supplementation daily during the 6-week preparatory period provided extra benefits for the power of the upper body as well as the strength of half squat and overhead press. However, 6 weeks of betaine supplementation had no effect on CMJ, bench-press strength, or sumo dead-lift strength.

Nobari et al. recruited professional soccer players to consume 2 g/day of betaine or placebo during a 14-week competition period, and the results showed that the performance on the countermovement jump (CMJ), 1-RM of bench press, and leg press significantly improved in both betaine and placebo groups [31]. Our findings indicate that 5 g of betaine supplementation daily during the 6-week preparatory period provided extra benefits than the 2 g/day of betaine supplementation for a 14-week competition period. A study indicated 14 days of betaine supplementation significantly increased the total repetitions and volume load in a 10-set bench-press protocol [27]; therefore, the enhancement contributed by betaine may promote the quality of subjects’ regular training in the preparatory period. Our results revealed that betaine supplementation effectively enhanced upper-body muscular power (as revealed by the OMBT test), and this result is consistent with Lee et al. [22]. However, some studies have demonstrated that betaine supplementation does not effectively enhance upper-body power [27,43]. The inconsistent results for upper-body power may be because participants in previous studies [27,43] did not maintain regular training during the experimental period, whereas participants in the present study and Lee et al.’s study [22] continued to train during the experimental period. Therefore, betaine supplementation combined with exercise training may benefit upper-body power. Moreover, because the OMBT and overhead press use similar muscle groups, such as the rotator cuff [44,45], the 1-RM overhead press in the participants of the betaine group was significantly enhanced after supplementation, which could be one of the reasons that betaine supplementation effectively increased the distance of the OMBT in the present study. Furthermore, our result that betaine supplementation did not enhance lower-body muscular power is consistent with Cholewa et al. [28]. The results indicated not only 2.5 g but also 5 g of betaine supplementation combined with training for 6 weeks would not enhance lower-body muscular power.

Our results regarding the effects of betaine supplementation would not increase 1-RM of bench press and sumo dead lift are not consistent with those of previous studies [22,29,43,46]. The inconsistent results may be due to the participants’ varying levels of activity. Our study provided the betaine supplement to the collegiate athletes in Division I, and they trained five times a week for sport-specific skills and twice a week for strength and conditioning. Differences between studies may be associated with methodological differences, including training status and/or conducted tests of muscular performance. Highly trained athletes have less potential for improvement after ergogenic aid supplementation, as their exercise performance and physical conditioning have reached their peak [47]. Our study also demonstrated that supplementing 5 g/day of betaine in collegiate athletes during the preparatory period provided extra benefits for the 1-RM of half squat and overhead press. Therefore, the supplementation strategy in the present study may be more effective than that in previous studies of 2–2.5 g/day [28,30,31]. The findings can be applied by strength and conditioning coaches for the training protocol in collegiate athletes. 

Betaine donates a methyl group to methylate phosphatidylethanolamine, forming phosphatidylcholine [48]. Studies have indicated that betaine supplementation improves the blood lipid profile [49,50] by increasing phosphatidylcholine synthesis and enhancing lipid transportation from the liver to the blood [51]. Moreover, because betaine is produced by the irreversible oxidation of choline, betaine supplementation does not deplete choline levels, resulting in the higher synthesis of phosphatidylcholine in the liver [20]. Furthermore, a systematic review and meta-analysis selected eight studies of betaine supplementation without exercise intervention and reported that betaine supplementation at a daily dosage of ≥4 g increased the concentrations of TC and LDL-C [52]. Our results revealed that 5 g/day of betaine supplementation combined with training for 6 weeks significantly decreased the TC concentration and had no negative effect on TG, LDL-C, or HDL-C. The present study demonstrated that 5 g/day of betaine supplementation in collegiate athletes of Division I during the preparatory period, including specific training and resistance training programs, had a positive effect on muscular performance and had no negative effect on blood lipid profiles. Previous research has demonstrated that 6–8 weeks of resistance training in healthy young male and female subjects can effectively reduce blood lipid levels [50,53]. Therefore, athletes can implement the supplementation strategy during the regular training season.

One limitation of this study is the relatively small sample due to the difficulty in recruiting other athletes to match the training arrangement (similar sport-specific skills training and strength and conditioning training program for 6 weeks), and some withdrew during the study due to acute musculoskeletal injuries. This could possibly increase the chance for type 2 errors; therefore, a larger-sample study might be needed in the future.

## 5. Conclusions

The present study provides novel and intriguing data that betaine supplementation (5 g/day for 6 weeks) in combination with sport-specific skills and strength and condition training significantly enhance OMBT distance and the 1-RM of half squat and overhead press in collegiate athletes. However, betaine supplementation had no effect on the 1-RM of bench press or sumo dead lift. The intervention in the study had no negative effect on blood lipid profiles. Future studies should focus on the dosage, days of supplementation, and training situation during supplementation for developing an effective and efficient supplementation strategy of betaine for enhancing athletes’ muscular performance. Betaine seems to be a useful nutritional strategy to improve and maintain performance during a 6-week preparatory periods in collegiate athletes.

## Figures and Tables

**Table 1:** Participant characteristics.

Variable	Betaine Group (n = 9)	Placebo Group (n = 7)
Age (years)	20.00 ± 1.94	20.00 ± 1.29
Body height (cm)	177.78 ± 5.49	173.00 ± 7.42
Body weight (kg)	72.81 ± 6.95	64.06 ± 9.21
Body fat percentage (%)	13.89 ± 3.69	11.97 ± 5.85

Data presented as means ± standard deviation.

**Table 2:** Changes in the blood lipid profile before and after 6 weeks of betaine supplementation.

Variable	Betaine (n = 7)	Placebo (n = 7)
Triglycerides (mg/dL)		
Pre	77.57 ± 27.98	83.71 ± 22.60
Post	66.71 ± 25.36	67.00 ± 35.34
Total cholesterol (mg/dL)		
Pre	201.71 ± 26.59	176.29 ± 20.88
Post	175.71 ± 30.28 *	151.00 ± 22.17 *
High-density lipoprotein cholesterol (mg/dL)		
Pre	62.29 ± 9.95	56.14 ± 12.28
Post	60.57 ± 11.54	54.00 ± 4.80
Low-density lipoprotein cholesterol (mg/dL)		
Pre	123.91 ± 28.04	103.40 ± 17.54
Post	101.80 ± 28.69	83.60 ± 25.69 *

Data are presented as means ± standard deviation. Pre: before supplementation; Post: after supplementation. *: Significant difference from Pre (*p* < 0.05).

## Data Availability

The data that support the findings of this study are available from the corresponding author upon reasonable request.
